# Supplementary material for: Modeling glioblastoma heterogeneity as a dynamic network of cell states
Source: Mol Syst Biol. 2021 Sep 16;17(9):e10105. doi: 10.15252/msb.202010105 (PMC8444284; doi:10.15252/msb.202010105)
Supplement: Supplementary file 5 — Source Data for Figure 3 [file MSB-17-e10105-s001.zip › Figure3A_sourcedata/GSEA_3065/nosigdb_state3.GseaPreranked.1622718426028/IVY_CT.html]

Details for gene set IVY\_CT[GSEA]

|  || Dataset | state3 |
| Phenotype | NoPhenotypeAvailable |
| Upregulated in class | na\_pos |
| GeneSet | IVY\_CT |
| Enrichment Score (ES) | 0.58766896 |
| Normalized Enrichment Score (NES) | 2.4269936 |
| Nominal p-value | 0.0 |
| FDR q-value | 0.0 |
| FWER p-Value | 0.0 |
Table: GSEA Results Summary

  

Fig 1: Enrichment plot: IVY\_CT      
 Profile of the Running ES Score & Positions of GeneSet Members on the Rank Ordered List

  

| PROBE | GENE SYMBOL | GENE\_TITLE | RANK IN GENE LIST | RANK METRIC SCORE | RUNNING ES | CORE ENRICHMENT || 1 | BCAN |  |  | 0 | 0.883 | 0.0418 | Yes |
| 2 | SCRG1 |  |  | 3 | 0.645 | 0.0721 | Yes |
| 3 | CENPF |  |  | 4 | 0.619 | 0.1014 | Yes |
| 4 | PTPRZ1 |  |  | 14 | 0.532 | 0.1256 | Yes |
| 5 | PTN |  |  | 22 | 0.493 | 0.1481 | Yes |
| 6 | ASPM |  |  | 25 | 0.480 | 0.1706 | Yes |
| 7 | TOP2A |  |  | 42 | 0.448 | 0.1900 | Yes |
| 8 | DSEL |  |  | 62 | 0.392 | 0.2063 | Yes |
| 9 | LPL |  |  | 68 | 0.381 | 0.2238 | Yes |
| 10 | NES |  |  | 113 | 0.332 | 0.2344 | Yes |
| 11 | SOX11 |  |  | 117 | 0.325 | 0.2494 | Yes |
| 12 | CENPE |  |  | 126 | 0.321 | 0.2637 | Yes |
| 13 | BCHE |  |  | 136 | 0.315 | 0.2775 | Yes |
| 14 | DNMT1 |  |  | 149 | 0.310 | 0.2908 | Yes |
| 15 | FADS2 |  |  | 153 | 0.309 | 0.3051 | Yes |
| 16 | IGFBP2 |  |  | 164 | 0.303 | 0.3183 | Yes |
| 17 | TTYH1 |  |  | 183 | 0.293 | 0.3301 | Yes |
| 18 | DLGAP5 |  |  | 207 | 0.279 | 0.3406 | Yes |
| 19 | PCDHGC3 |  |  | 228 | 0.272 | 0.3511 | Yes |
| 20 | SMC4 |  |  | 265 | 0.255 | 0.3590 | Yes |
| 21 | NOVA1 |  |  | 270 | 0.253 | 0.3705 | Yes |
| 22 | COL9A3 |  |  | 278 | 0.250 | 0.3816 | Yes |
| 23 | VCAN |  |  | 293 | 0.245 | 0.3916 | Yes |
| 24 | EDNRB |  |  | 305 | 0.242 | 0.4017 | Yes |
| 25 | MATN2 |  |  | 318 | 0.239 | 0.4117 | Yes |
| 26 | SEMA6A |  |  | 387 | 0.222 | 0.4143 | Yes |
| 27 | KIF15 |  |  | 428 | 0.213 | 0.4197 | Yes |
| 28 | FREM2 |  |  | 431 | 0.212 | 0.4295 | Yes |
| 29 | DDR1 |  |  | 454 | 0.208 | 0.4368 | Yes |
| 30 | ITGB8 |  |  | 463 | 0.206 | 0.4457 | Yes |
| 31 | CREB5 |  |  | 497 | 0.199 | 0.4512 | Yes |
| 32 | SDC3 |  |  | 539 | 0.194 | 0.4556 | Yes |
| 33 | TPX2 |  |  | 544 | 0.193 | 0.4643 | Yes |
| 34 | NLGN3 |  |  | 558 | 0.191 | 0.4719 | Yes |
| 35 | LRRN3 |  |  | 574 | 0.189 | 0.4791 | Yes |
| 36 | IGF2BP3 |  |  | 597 | 0.186 | 0.4853 | Yes |
| 37 | NCAPG |  |  | 601 | 0.186 | 0.4938 | Yes |
| 38 | OLIG1 |  |  | 602 | 0.186 | 0.5026 | Yes |
| 39 | SCD5 |  |  | 619 | 0.184 | 0.5094 | Yes |
| 40 | NUF2 |  |  | 680 | 0.174 | 0.5107 | Yes |
| 41 | CSPG5 |  |  | 716 | 0.168 | 0.5146 | Yes |
| 42 | BUB1 |  |  | 717 | 0.168 | 0.5225 | Yes |
| 43 | BLM |  |  | 811 | 0.156 | 0.5191 | Yes |
| 44 | CRISPLD1 |  |  | 840 | 0.154 | 0.5232 | Yes |
| 45 | GTSE1 |  |  | 852 | 0.152 | 0.5291 | Yes |
| 46 | BUB1B |  |  | 857 | 0.152 | 0.5358 | Yes |
| 47 | KIF14 |  |  | 862 | 0.151 | 0.5425 | Yes |
| 48 | CDK1 |  |  | 915 | 0.147 | 0.5434 | Yes |
| 49 | CLSPN |  |  | 922 | 0.146 | 0.5497 | Yes |
| 50 | ETV1 |  |  | 963 | 0.141 | 0.5517 | Yes |
| 51 | WSCD1 |  |  | 978 | 0.140 | 0.5567 | Yes |
| 52 | DLL3 |  |  | 999 | 0.138 | 0.5609 | Yes |
| 53 | LMNB1 |  |  | 1021 | 0.137 | 0.5650 | Yes |
| 54 | MLC1 |  |  | 1033 | 0.136 | 0.5701 | Yes |
| 55 | NDC80 |  |  | 1087 | 0.131 | 0.5702 | Yes |
| 56 | FANCI |  |  | 1092 | 0.131 | 0.5759 | Yes |
| 57 | MEX3A |  |  | 1101 | 0.130 | 0.5811 | Yes |
| 58 | TTK |  |  | 1177 | 0.123 | 0.5782 | Yes |
| 59 | RAD51AP1 |  |  | 1196 | 0.122 | 0.5819 | Yes |
| 60 | MCM10 |  |  | 1197 | 0.122 | 0.5877 | Yes |
| 61 | NUSAP1 |  |  | 1338 | 0.113 | 0.5767 | No |
| 62 | ESCO2 |  |  | 1373 | 0.110 | 0.5780 | No |
| 63 | CDCA7 |  |  | 1440 | 0.106 | 0.5754 | No |
| 64 | PBK |  |  | 1524 | 0.102 | 0.5705 | No |
| 65 | FAM181B |  |  | 1629 | 0.096 | 0.5630 | No |
| 66 | LRRN1 |  |  | 1690 | 0.092 | 0.5604 | No |
| 67 | C1orf61 |  |  | 1703 | 0.091 | 0.5633 | No |
| 68 | OLIG2 |  |  | 1742 | 0.089 | 0.5631 | No |
| 69 | SPATA6 |  |  | 1796 | 0.087 | 0.5611 | No |
| 70 | KIF2C |  |  | 1985 | 0.077 | 0.5429 | No |
| 71 | PLP1 |  |  | 2047 | 0.074 | 0.5393 | No |
| 72 | UHRF1 |  |  | 2136 | 0.071 | 0.5324 | No |
| 73 | LRRC17 |  |  | 2223 | 0.067 | 0.5256 | No |
| 74 | CDCA3 |  |  | 2232 | 0.067 | 0.5279 | No |
| 75 | CDCA8 |  |  | 2281 | 0.065 | 0.5254 | No |
| 76 | ID4 |  |  | 2306 | 0.064 | 0.5256 | No |
| 77 | EGFR |  |  | 2416 | 0.060 | 0.5158 | No |
| 78 | CCNE2 |  |  | 2692 | 0.050 | 0.4862 | No |
| 79 | DTL |  |  | 2697 | 0.050 | 0.4882 | No |
| 80 | SHOX2 |  |  | 2699 | 0.050 | 0.4904 | No |
| 81 | NLGN1 |  |  | 2733 | 0.049 | 0.4889 | No |
| 82 | RRM2 |  |  | 2835 | 0.046 | 0.4794 | No |
| 83 | NPAS3 |  |  | 2988 | 0.041 | 0.4637 | No |
| 84 | ABAT |  |  | 3059 | 0.040 | 0.4574 | No |
| 85 | TCF12 |  |  | 3107 | 0.038 | 0.4538 | No |
| 86 | KIF4A |  |  | 3195 | 0.036 | 0.4454 | No |
| 87 | POU3F2 |  |  | 3388 | 0.031 | 0.4245 | No |
| 88 | FAM111B |  |  | 3448 | 0.029 | 0.4191 | No |
| 89 | MELK |  |  | 3462 | 0.029 | 0.4189 | No |
| 90 | SOX8 |  |  | 3470 | 0.029 | 0.4195 | No |
| 91 | CCNB2 |  |  | 3502 | 0.028 | 0.4172 | No |
| 92 | AURKA |  |  | 3503 | 0.028 | 0.4185 | No |
| 93 | SRPX |  |  | 3518 | 0.028 | 0.4182 | No |
| 94 | SAPCD2 |  |  | 3698 | 0.023 | 0.3985 | No |
| 95 | NXPH1 |  |  | 3773 | 0.021 | 0.3909 | No |
| 96 | CKAP2L |  |  | 3787 | 0.020 | 0.3903 | No |
| 97 | SALL1 |  |  | 3800 | 0.020 | 0.3899 | No |
| 98 | PRC1 |  |  | 3878 | 0.018 | 0.3818 | No |
| 99 | NRM |  |  | 3973 | 0.016 | 0.3716 | No |
| 100 | DCLK2 |  |  | 4033 | 0.014 | 0.3655 | No |
| 101 | CDCA2 |  |  | 4066 | 0.013 | 0.3624 | No |
| 102 | RAMP1 |  |  | 4100 | 0.013 | 0.3592 | No |
| 103 | FBXO5 |  |  | 4119 | 0.012 | 0.3576 | No |
| 104 | TACC3 |  |  | 4146 | 0.011 | 0.3551 | No |
| 105 | GAS1 |  |  | 4210 | 0.009 | 0.3483 | No |
| 106 | CENPK |  |  | 4433 | 0.003 | 0.3226 | No |
| 107 | SOX9 |  |  | 4503 | 0.001 | 0.3147 | No |
| 108 | MSI1 |  |  | 4661 | -0.003 | 0.2966 | No |
| 109 | RFX4 |  |  | 4672 | -0.003 | 0.2956 | No |
| 110 | FIBIN |  |  | 4788 | -0.006 | 0.2825 | No |
| 111 | LFNG |  |  | 4861 | -0.007 | 0.2745 | No |
| 112 | EZH2 |  |  | 4952 | -0.010 | 0.2645 | No |
| 113 | ASF1B |  |  | 5007 | -0.011 | 0.2587 | No |
| 114 | SOX21 |  |  | 5077 | -0.013 | 0.2513 | No |
| 115 | FERMT1 |  |  | 5185 | -0.015 | 0.2396 | No |
| 116 | HMGB2 |  |  | 5479 | -0.023 | 0.2067 | No |
| 117 | KCNQ2 |  |  | 5701 | -0.030 | 0.1825 | No |
| 118 | HES6 |  |  | 5805 | -0.033 | 0.1721 | No |
| 119 | CDC20 |  |  | 5818 | -0.033 | 0.1723 | No |
| 120 | CENPM |  |  | 5855 | -0.034 | 0.1697 | No |
| 121 | GATM |  |  | 6161 | -0.045 | 0.1364 | No |
| 122 | HIST1H1D |  |  | 6428 | -0.054 | 0.1081 | No |
| 123 | METTL7B |  |  | 6482 | -0.056 | 0.1046 | No |
| 124 | KLHDC8A |  |  | 7407 | -0.109 | 0.0024 | No |
| 125 | NMB |  |  | 7495 | -0.116 | -0.0022 | No |
| 126 | GINS2 |  |  | 7545 | -0.122 | -0.0021 | No |
| 127 | DHFR |  |  | 7593 | -0.127 | -0.0015 | No |
| 128 | CDKN2C |  |  | 7609 | -0.128 | 0.0028 | No |
| 129 | TSC22D4 |  |  | 7610 | -0.129 | 0.0089 | No |
| 130 | CKB |  |  | 7659 | -0.134 | 0.0097 | No |
| 131 | BIRC5 |  |  | 7735 | -0.144 | 0.0078 | No |
| 132 | MYBL2 |  |  | 7771 | -0.148 | 0.0107 | No |
| 133 | HIST1H1B |  |  | 7908 | -0.168 | 0.0029 | No |
| 134 | PCBP4 |  |  | 8002 | -0.186 | 0.0009 | No |
| 135 | ATP6V0E2 |  |  | 8025 | -0.189 | 0.0073 | No |
| 136 | KCNIP1 |  |  | 8244 | -0.238 | -0.0068 | No |
| 137 | PTTG1 |  |  | 8366 | -0.279 | -0.0076 | No |
| 138 | ID3 |  |  | 8502 | -0.341 | -0.0071 | No |
| 139 | METRN |  |  | 8563 | -0.374 | 0.0036 | No |
| 140 | DBI |  |  | 8571 | -0.383 | 0.0209 | No |
Table: GSEA details [plain text format]

  

Fig 2: IVY\_CT: Random ES distribution      
 Gene set null distribution of ES for **IVY\_CT**
